# Association of Congenital and Acquired Cardiovascular Conditions With COVID-19 Severity Among Pediatric Patients in the US

**DOI:** 10.1001/jamanetworkopen.2022.11967

**Published:** 2022-05-17

**Authors:** Louis Ehwerhemuepha, Bradley Roth, Anita K. Patel, Olivia Heutlinger, Carly Heffernan, Antonio C. Arrieta, Terence Sanger, Dan M. Cooper, Babak Shahbaba, Anthony C. Chang, William Feaster, Sharief Taraman, Hiroki Morizono, Rachel Marano

**Affiliations:** 1 Children’s Health of Orange County, Orange, California; 2 University of California-Irvine School of Medicine, Irvine; 3 Children’s National Hospital System and George Washington University School of Medicine and Health Sciences, Washington, DC

## Abstract

**Importance:**

Identifying the associations between severe COVID-19 and individual cardiovascular conditions in pediatric patients may inform treatment.

**Objective:**

To assess the association between previous or preexisting cardiovascular conditions and severity of COVID-19 in pediatric patients.

**Design, Setting, and Participants:**

This retrospective cohort study used data from a large, multicenter, electronic health records database in the US. The cohort included patients aged 2 months to 17 years with a laboratory-confirmed diagnosis of COVID-19 or a diagnosis code indicating infection or exposure to SARS-CoV-2 at 85 health systems between March 1, 2020, and January 31, 2021.

**Exposures:**

Diagnoses for 26 cardiovascular conditions between January 1, 2015, and December 31, 2019 (before infection with SARS-CoV-2).

**Main Outcomes and Measures:**

The main outcome was severe COVID-19, defined as need for supplemental oxygen or in-hospital death. Mixed-effects, random intercept logistic regression modeling assessed the significance and magnitude of associations between 26 cardiovascular conditions and COVID-19 severity. Multiple comparison adjustment was performed using the Benjamini-Hochberg false discovery rate procedure.

**Results:**

The study comprised 171 416 pediatric patients; the median age was 8 years (IQR, 2-14 years), and 50.28% were male. Of these patients, 17 065 (9.96%) had severe COVID-19. The random intercept model showed that the following cardiovascular conditions were associated with severe COVID-19: cardiac arrest (odds ratio [OR], 9.92; 95% CI, 6.93-14.20), cardiogenic shock (OR, 3.07; 95% CI, 1.90-4.96), heart surgery (OR, 3.04; 95% CI, 2.26-4.08), cardiopulmonary disease (OR, 1.91; 95% CI, 1.56-2.34), heart failure (OR, 1.82; 95% CI, 1.46-2.26), hypotension (OR, 1.57; 95% CI, 1.38-1.79), nontraumatic cerebral hemorrhage (OR, 1.54; 95% CI, 1.24-1.91), pericarditis (OR, 1.50; 95% CI, 1.17-1.94), simple biventricular defects (OR, 1.45; 95% CI, 1.29-1.62), venous embolism and thrombosis (OR, 1.39; 95% CI, 1.11-1.73), other hypertensive disorders (OR, 1.34; 95% CI, 1.09-1.63), complex biventricular defects (OR, 1.33; 95% CI, 1.14-1.54), and essential primary hypertension (OR, 1.22; 95% CI, 1.08-1.38). Furthermore, 194 of 258 patients (75.19%) with a history of cardiac arrest were younger than 12 years.

**Conclusions and Relevance:**

The findings suggest that some previous or preexisting cardiovascular conditions are associated with increased severity of COVID-19 among pediatric patients in the US and that morbidity may be increased among individuals children younger than 12 years with previous cardiac arrest.

## Key Points

**Question:**

What is the association between individual congenital and acquired cardiovascular conditions and COVID-19 severity in pediatric patients?

**Findings:**

In this cohort study of 171 416 US individuals aged 2 months to 17 years with SARS-CoV-2 infection, cardiac arrest, cardiogenic shock, heart surgery, cardiopulmonary disease, heart failure, hypotension, nontraumatic cerebral hemorrhage, pericarditis, and biventricular defects were associated with increased COVID-19 severity.

**Meaning:**

The findings suggest that previous or preexisting cardiovascular conditions are associated with increased COVID-19 severity, in varying degrees, in pediatric patients.

## Teaser

This cohort study evaluates the association of congenital and acquired cardiovascular conditions with severity of COVID-19 among pediatric patients in the US.

## Introduction

The novel SARS-CoV-2 continues to be a threat to global health. Severe illness from this virus occurs in children, although not as frequently as in adults. More than 5 million children have tested positive for SARS-CoV-2 to date,^1^ with 0.2% to 4.4% of cases resulting in hospitalization.^2,3^ In adults, preexisting cardiovascular conditions have been implicated in severe COVID-19 and are associated with increased morbidity and mortality.^4,5,6,7,8,9,10,11^ An early meta-analysis of COVID-19 cases in China found an association between cardiovascular conditions and unfavorable clinical outcomes.^4,7,12^ COVID-19 has been shown to be associated with development of cardiovascular conditions, including myocardial injury, arrhythmia, acute coronary syndrome, and venous thromboembolism.^5,6,10,11,13,14,15,16,17^

Although early data relating to pediatric COVID-19 cases are limited, systematic reviews of cases in children showed that underlying cardiovascular defects were prevalent, with congenital heart disease (CHD) being associated with worse clinical outcomes.^18,19^ Congenital heart disease is estimated to occur in 3 to 10 children per 1000 live births in the US,^20,21,22^ and this rate is increasing.^23^ Therefore, a substantial number of children are affected by congenital and acquired cardiovascular conditions.

In this study, we assessed the association of previous or preexisting cardiovascular conditions with the severity of COVID-19 among pediatric patients. Identification of risk factors and estimation of corresponding odds ratios (ORs) may provide useful information for management of these conditions in pediatric patients infected with SARS-CoV-2. Previous studies have shown that preexisting conditions may be associated with increased severity of COVID-19 in children,^8,24^ but little is known about the associations of individual cardiovascular conditions with odds of increased COVID-19 severity. The objective of this study was to evaluate cardiovascular factors associated with severe COVID-19 in pediatrics and to estimate corresponding ORs. We hypothesized that there would be substantial differences in the magnitude of the ORs among cardiovascular factors associated with severe COVID-19 that may provide clinically useful information for treatment of these patients.

## Methods

This retrospective cohort study was approved by the institutional review board of Children’s Health of Orange County. Informed consent requirement was waived by this institutional review board because the database used was deidentified and the study was not classified as human subject research and posed no more than minimal risk to patients. We followed the Strengthening the Reporting of Observational Studies in Epidemiology (STROBE) reporting guideline.

### Data Source

Cerner Real-World Data, a large, multicenter electronic health records database, was used for this study. As of August 2021, Cerner Real-World Data housed data from 110 health systems and more than 1.2 billion clinical encounters from all care settings in the US. This clinical database is powered by Cerner HealtheIntent, an electronic health records–agnostic and insights platform.^25^ HealtheIntent retrieves data from the electronic health records of individual health systems. These data are combined across health systems and then deidentified, encrypted, and secured in compliance with the privacy regulation of the Health Insurance Portability and Accountability Act of 1996 (eFigure in the Supplement provides a geographical distribution of patients from all health systems contributing to the database). In this study, we used data on COVID-19−related encounters from 85 health systems between March 1, 2020, and January 31, 2021. Previous or preexisting cardiovascular and chronic conditions (before infection with SARS-CoV-2) were ascertained through retrieval of diagnoses between January 1, 2015, and December 31, 2019, for qualified patients.

### Patients and Variables

The study cohort consisted of patients between 2 months and 17 years of age who had a laboratory-confirmed diagnosis of COVID-19 or a diagnosis code indicating infection or exposure to SARS-CoV-2. Only the first COVID-19−related encounter was considered in cases of multiple encounters for COVID-19 per patient. Neonates and infants younger than 2 months were excluded because of insufficient medical history (Figure).

**Figure.**
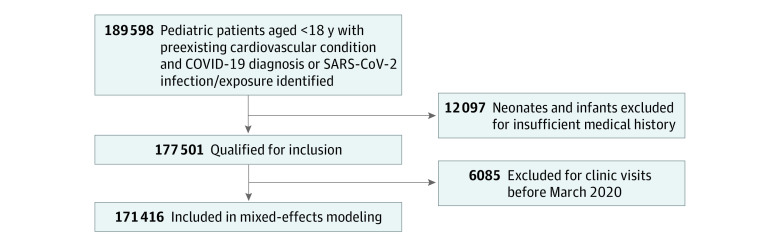
Inclusion and Exclusion Criteria for the Study Sample

Twenty-six previous or preexisting cardiovascular conditions were identified using *International Statistical Classification of Diseases, Tenth Revision, Clinical Modification (ICD-10-CM)* codes, for a comprehensive list of congenital and acquired conditions affecting the cardiovascular system. These conditions included single ventricle defects, simple biventricular defects (BVDs), and complex BVDs as classes of CHD^26,27^ as well as cardiac arrest, cardiogenic shock, heart surgery, and 20 other acquired conditions. Definitions for the 3 classes of CHD are shown in the eTable in the Supplement.^27^ A complete list of all cardiovascular conditions considered is shown in Table 1.

**Table 1.** Summary Statistics and Univariable Analyses

Variable	Patients, No. (%) (N = 171 416)		Unadjusted odds ratio (95% CI)
	Mild COVID-19 (n = 154 351)	Severe COVID-19 (n = 17 065)^a^	
**Demographic characteristics **			
Age			
3-12 mo	15 193 (9.84)	1692 (9.92)	0.96 (0.91-1.02)
13 mo to 2 y	15 720 (10.18)	1401 (8.21)	0.77 (0.72-0.82)
3-5 y	25 227 (16.34)	2682 (15.72)	0.92 (0.88-0.96)
6-11 y	38 552 (24.98)	4376 (25.64)	0.98 (0.94-1.02)
12-17 y	44 435 (22.34)	4582 (21.15)	1 [Reference]
Sex			
Female	77 439 (50.17)	7381 (43.25)	1 [Reference]
Male	76 542 (49.59)	9639 (56.48)	1.32 (1.28-1.36)
Unknown	370 (0.24)	45 (0.26)	1.28 (0.92-1.72)
Race			
American Indian or Alaska Native	1918 (1.24)	501 (2.94)	2.49 (2.25-2.75)
Asian or Pacific Islander	2385 (1.55)	426 (2.50)	1.70 (1.53-1.89)
Black or African American	22 461 (14.55)	2309 (13.53)	0.98 (0.94-1.03)
White	99 362 (64.37)	10 417 (61.04)	1 [Reference]
Mixed	1239 (0.80)	233 (1.37)	1.79 (1.55-2.06)
Other^b^	12 308 (7.97)	1559 (9.14)	1.21 (1.14-1.28)
Unknown	14 678 (9.51)	1620 (9.49)	1.05 (1.00-1.11)
Ethnicity			
Hispanic or Latino	69 813 (45.23)	6260 (36.68)	0.67 (0.65-0.69)
Not Hispanic or Latino	75 269 (48.76)	10 081 (59.07)	1 [Reference]
Unknown	9269 (6.01)	724 (4.24)	0.58 (0.54-0.63)
**Payer**			
Governmental or public insurance	82 171 (53.24)	7852 (46.01)	1 [Reference]
Nongovernmental or private insurance	25 742 (16.68)	3031 (17.76)	1.23 (1.18-1.29)
Unknown	46 438 (30.09)	6182 (36.23)	1.39 (1.34-1.44)
**Encounter type**			
Inpatient	14 130 (9.15)	10 508 (61.58)	1 [Reference]
Observation	6410 (4.15)	4341 (25.44)	0.91 (0.87-0.95)
Emergency	101 580 (65.81)	2127 (12.46)	0.03 (0.03-0.03)
Urgent care	32 231 (20.88)	89 (0.52)	0.004 (0.003-0.01)
Treated for cardiovascular condition during COVID-19–related encounter			
No	154 146 (99.87)	16 880 (98.92)	1 [Reference]
Yes	205 (0.13)	185 (1.08)	8.24 (6.75-10.06)
**Previous or preexisting cardiovascular conditions** ^c^			
Heart surgery			
No	154 223 (99.92)	16 905 (99.06)	1 [Reference]
Yes	128 (0.08)	160 (0.94)	11.4 (9.04-14.41)
Vascular surgery			
No	150 810 (97.71)	16 254 (95.25)	1 [Reference]
Yes	3541 (2.29)	811 (4.75)	2.13 (1.96-2.30)
Single ventricle defects			
No	154 174 (99.89)	16 898 (99.02)	1 [Reference]
Yes	177 (0.11)	167 (0.98)	8.61 (6.96-10.64)
Simple BVDs			
No	152 376 (98.72)	16 014 (93.84)	1 [Reference]
Yes	1975 (1.28)	1051 (6.16)	5.06 (4.69-5.47)
Complex BVDs			
No	153 197 (99.25)	16 337 (95.73)	1 [Reference]
Yes	1154 (0.75)	728 (4.27)	5.92 (5.38-6.50)
Cardiogenic shock (R57.0)			
No	154 314 (99.98)	16 978 (99.49)	1 [Reference]
Yes	37 (0.02)	87 (0.51)	21.37 (14.67-31.76)
Chronic rheumatic heart disease (I05-I09)			
No	154 192 (99.90)	16 936 (99.24)	1 [Reference]
Yes	159 (0.10)	129 (0.76)	7.39 (5.85-9.32)
Essential primary hypertension (I10)			
No	152 937 (99.08)	16 326 (95.67)	1 [Reference]
Yes	1414 (0.92)	739 (4.33)	4.90 (4.47-5.36)
Other hypertensive heart disease (I11-I16)			
No	153 997 (99.77)	16 758 (98.20)	1 [Reference]
Yes	354 (0.23)	307 (1.80)	7.97 (6.83-9.29)
Ischemic heart disease (I20-I25)			
No	154 267 (99.95)	17 000 (99.62)	1 [Reference]
Yes	84 (0.05)	65 (0.38)	7.02 (5.06-9.70)
Pulmonary heart disease (I26-I27)			
No	154 099 (99.84)	16 699 (97.86)	1 [Reference]
Yes	252 (0.16)	366 (2.14)	13.4 (11.41-15.76)
Pericarditis (I30-I32)			
No	154 155 (99.87)	16 886 (98.95)	1 [Reference]
Yes	196 (0.13)	179 (1.05)	8.34 (6.80-10.22)
Endocarditis (I33-I39)			
No	153 859 (99.68)	16 728 (98.03)	1 [Reference]
Yes	492 (0.32)	337 (1.97)	6.30 (5.48-7.24)
Cardiomyopathy (I42-I43)			
No	154 183 (99.89)	16 931 (99.21)	1 [Reference]
Yes	168 (0.11)	134 (0.79)	7.26 (5.78-9.11)
Atrioventricular and other conduction blocks (I44-I45)			
No	153 918 (99.72)	16 861 (98.80)	1 [Reference]
Yes	433 (0.28)	204 (1.20)	4.30 (3.63-5.08)
Cardiac arrest (I46)			
No	154 298 (99.97)	16 860 (98.80)	1 [Reference]
Yes	53 (0.03)	205 (1.20)	35.40 (26.38-48.36)
Arrhythmias (I47-I49)			
No	153 424 (99.40)	16 613 (97.35)	1 [Reference]
Yes	927 (0.60)	452 (2.65)	4.50 (4.02-5.04)
Heart failure (I50)			
No	154 049 (99.80)	16 709 (97.91)	1 [Reference]
Yes	302 (0.20)	356 (2.09)	10.87 (9.32-12.68)
Nontraumatic cerebral hemorrhage (I60-I62)			
No	154 049 (99.80)	16 845 (98.71)	1 [Reference]
Yes	302 (0.20)	220 (1.29)	6.66 (5.59-7.93)
Cerebral infarction (I63)			
No	154 227 (99.92)	16 969 (99.44)	1 [Reference]
Yes	124 (0.08)	96 (0.56)	7.04 (5.38-9.18)
Arterial embolism and thrombosis (I74)			
No	154 308 (99.97)	17 010 (99.68)	1 [Reference]
Yes	43 (0.03)	55 (0.32)	11.6 (7.80-17.38)
Phlebitis and thrombophlebitis (I80)			
No	154 303 (99.97)	17 025 (99.77)	1 [Reference]
Yes	48 (0.03)	40 (0.23)	7.55 (4.94-11.48)
Venous embolism and thrombosis (I81-I82)			
No	154 077 (99.82)	16 775 (98.30)	1 [Reference]
Yes	274 (0.18)	290 (1.70)	9.72 (8.24-11.48)
Disorder of lymphatic vessels and lymph nodes (I88-I89)			
No	152 897 (99.06)	16 848 (98.73)	1 [Reference]
Yes	1454 (0.94)	217 (1.27)	1.35 (1.17-1.56)
Hypotension (I95)			
No	153 407 (99.39)	16 424 (96.24)	1 [Reference]
Yes	944 (0.61)	641 (3.76)	6.34 (5.73-7.02)
Intraoperative and postprocedural complications (I97)			
No	154 279 (99.95)	16 972 (99.46)	1 [Reference]
Yes	72 (0.05)	93 (0.54)	11.74 (8.64-16.02)
**Complex chronic conditions**			
Respiratory			
No	151 800 (98.35)	15 992 (93.71)	1 [Reference]
Yes	2551 (1.65)	1073 (6.29)	3.99 (3.71-4.29)
Neurologic			
No	151 023 (97.84)	15 071 (88.32)	1 [Reference]
Yes	3328 (2.16)	1994 (11.68)	6.00 (5.67-6.36)
Hematologic or immunologic			
No	151 584 (98.21)	16 009 (93.81)	1 [Reference]
Yes	2767 (1.79)	1056 (6.19)	3.61 (3.36-3.88)
Gastrointestinal			
No	152 917 (99.07)	15 487 (90.75)	1 [Reference]
Yes	1434 (0.93)	1578 (9.25)	10.87 (10.10-11.69)
Metabolic			
No	149 477 (96.84)	15 604 (91.44)	1 [Reference]
Yes	4874 (3.16)	1461 (8.56)	2.87 (2.70-3.05)
Malignant neoplasm			
No	153 329 (99.34)	16 479 (96.57)	1 [Reference]
Yes	1022 (0.66)	586 (3.43)	5.34 (4.81-5.91)
Renal or urologic			
No	152 475 (98.78)	15 878 (93.04)	1 [Reference]
Yes	1876 (1.22)	1187 (6.96)	6.08 (5.64-6.54)
Congenital or genetic			
No	151 166 (97.94)	15 573 (91.26)	1 [Reference]
Yes	3185 (2.06)	1492 (8.74)	4.55 (4.27-4.85)
Obesity			
No	135 694 (87.91)	13 306 (77.97)	1 [Reference]
Yes	18 657 (12.09)	3759 (22.03)	2.05 (1.98-2.14)

Abbreviation: BVDs, biventricular defects.

^a^ COVID-19 was classified as severe if supplemental oxygen was needed or if death occurred.

^b^ Other was not categorized.

^c^ Codes in parentheses are *International Statistical Classification of Diseases, Tenth Revision, Clinical Modification* codes.

We also retrieved potential confounders, such as demographic characteristics; categories of pediatric complex chronic conditions, as defined by Feudtner et al,^28,29^ using diagnosis and procedure codes; comorbidities such as obesity; and indicators of whether the patient was treated for a cardiovascular condition concomitantly with COVID-19 diagnosis during the index encounter. Obesity was defined as a body mass index (BMI) at the 95th percentile or greater for patients with BMI on record and as *ICD-10-CM* diagnosis codes E66.0 and E66.9 for those whose medical records had incomplete information. Patient age was categorized using definitions from the Eunice Kennedy Shriver National Institute of Child Health and Human Development.^30^

A binary outcome variable of mild vs severe COVID-19 was defined. Patients who did not receive oxygen therapy and who were discharged to home alive were defined as having had mild COVID-19, whereas those who received oxygen therapy or died in the hospital were defined as having had severe COVID-19. Need for oxygen therapy was determined based on dependence on nasal cannula, high-flow nasal cannula, bilevel or continuous positive airway pressure machine, mechanical ventilation, or extracorporeal membrane oxygenation; this definition is consistent with published literature.^31,32,33^ Use of supplemental oxygen therapy was identified using corresponding Healthcare Common Procedure Coding System codes as well as clinical events during the encounter.

### Statistical Analysis

Missing data were addressed by coding a nuisance level or category for sex, race, and ethnicity. (Race and ethnicity were likely self-reported by patients or their legal guardians.) Only patients with a recorded date of birth (age) were considered because the database includes patients of all ages. Absence of an *ICD-10-CM* diagnosis code or medication on record for a patient was viewed as indicative of an inapplicable condition or treatment choice for the patient. Information on confirmed laboratory markers of organ dysfunction was provided as descriptive statistics for insights into other complications that were outside the scope of the definition for severe COVID-19 used in this study.

A random intercept (mixed-effects) logistic regression model was used to assess the association between COVID-19 severity and each of the 26 cardiovascular conditions examined while controlling for demographics, payer, complex chronic conditions, and indicators of whether a cardiovascular condition was treated concomitantly with COVID-19 during the index encounter. Data on the health systems that provided care to patients were used as the random intercept to account for spatial structure and baseline clinical differences between health systems. This indicator for treatment of a cardiovascular condition controlled for confounding given the need for oxygen therapy for a cardiovascular condition that may not have been associated with SARS-CoV-2 infection. Multiple-comparison adjustment was performed using the Benjamini-Hochberg false discovery rate procedure because of the large number of variables (and hypotheses) that were tested.^34^

Benjamini-Hochberg adjusted *P* values were used to determine significance at a threshold of 0.05. Statistical analyses were conducted using HealtheDataLab (Cerner) for parallel distributed cloud computing and R, version 4.1 (R Foundation for Statistical Computing).

## Results

A total of 171 416 patients from 85 health systems met the inclusion criteria. Of these patients, 154 351 (90.04%) had mild COVID-19 and 17 065 (9.96%) had severe COVID-19. In the overall cohort, 1.41% of patients were American Indian or Alaska Native; 1.64% were Asian or Pacific Islander; 14.45% were Black or African American; 44.38% were Hispanic or Latino; 64.04% were White; and 18.46% were from mixed, other, or unknown racial groups. A total of 49.48% of patients were female, 50.28% were male, and 0.24% had missing or unknown sex. The median age for all patients was 8 years (IQR, 2-14 years).

A median of 422 patients (IQR, 62-1268 patients) were contributed from each health system. The care settings of these encounters included emergency departments (60.50%), inpatient facilities (20.65%), and urgent care facilities (18.85%). Data on primary care clinic and telehealth visits were not available.

The study population included 8544 patients (4.98%) requiring a nasal cannula, 170 (0.10%) receiving bilevel or continuous positive airway pressure, 11 500 (6.71%) requiring a high-flow nasal cannula, 4116 (2.40%) receiving mechanical ventilation, and 29 (0.02%) receiving extracorporeal membrane oxygenation; 197 patients (0.11%) died. Overlap between these groups used to define severe COVID-19 resulted in 17 065 severe cases (9.96%). Data on BMI were found for 67.29% of patients; *ICD-10-CM* diagnosis codes were scanned for the remaining 32.71%. We estimated that less than 1% of patients may have had multisystem inflammatory syndrome in children (MIS-C) during the first COVID-19–related encounter because 0.34% of these patients received intravenous immunoglobulin therapy or a biologic agent such as rituximab or tocilizumab, as shown in Table 2.

**Table 2.** Medications, Complications, and Organ Dysfunction According to Severity of COVID-19

Variable	Patients With COVID-19, No. (%)	
	Mild (n = 154 351)	Severe (n = 17 065)^a^
**Medications**		
Remdesivir		
No	154 340 (99.99)	16 990 (99.56)
Yes	11 (0.01)	75 (0.44)
COVID-19 convalescent plasma		
No	154 350 (100)	17 063 (99.99)
Yes	1 (0)	2 (0.01)
Inotropes		
No	153 248 (99.29)	15 370 (90.07)
Yes	1103 (0.71)	1695 (9.93)
Dexamethasone		
No	148 595 (96.27)	8873 (52.00)
Yes	5756 (3.73)	8192 (48.00)
Enoxaparin		
No	154 034 (99.79)	16 128 (94.51)
Yes	317 (0.21)	937 (5.49)
Heparin		
No	154 166 (99.88)	15 989 (93.69)
Yes	185 (0.12)	1076 (6.31)
IVIG therapy		
No	154 086 (99.83)	16 818 (98.55)
Yes	265 (0.17)	247 (1.45)
Methylprednisolone		
No	153 624 (99.53)	15 578 (91.29)
Yes	727 (0.47)	1487 (8.71)
Rituximab		
No	154 334 (99.99)	17 026 (99.77)
Yes	17 (0.01)	39 (0.23)
Tocilizumab		
No	154 344 (100)	17 045 (99.88)
Yes	7 (0)	20 (0.12)
Aspirin		
No	153 876 (99.69)	16 469 (96.51)
Yes	475 (0.31)	596 (3.49)
**Complications and markers of organ dysfunction**		
Delirium		
No	154 145 (99.87)	16 987 (99.54)
Yes	206 (0.13)	78 (0.46)
Acute kidney injury (elevated creatinine level)		
No	153 505 (99.45)	16 419 (96.21)
Yes	846 (0.55)	646 (3.79)
PT		
Normal	153 650 (99.55)	15 607 (91.46)
High	701 (0.45)	1458 (8.54)
PT/INR		
Normal	153 798 (99.64)	15 886 (93.09)
High	553 (0.36)	1179 (6.91)
Aspartate aminotransferase level		
Normal	151 248 (97.99)	14 882 (87.21)
High	3103 (2.01)	2183 (12.79)
Alanine aminotransferase level		
Normal	150 877 (97.75)	15 095 (88.46)
High	3474 (2.25)	1970 (11.54)
Gamma-glutamyl transferase level		
Normal	154 167 (99.88)	16 789 (98.38)
High	184 (0.12)	276 (1.62)
Bilirubin level		
Normal	153 041 (99.15)	16 236 (95.14)
High	1310 (0.85)	829 (4.86)
PaO_2_ to FiO_2_ levels <100		
No	154 349 (100)	16 957 (99.37)
Yes	2 (0)	108 (0.63)
Viral pneumonia		
No	153 870 (99.69)	16 675 (97.71)
Yes	481 (0.31)	390 (2.29)

Abbreviations: FiO_2_, fraction of inspired oxygen; INR, international normalized ratio; IVIG, intravenous immunoglobulin; PaO_2_, partial pressure of oxygen in arterial blood; PT, prothrombin time.

^a^ COVID-19 was classified as severe if supplemental oxygen was needed or if death occurred.

Summary statistics and univariable (unadjusted) statistics are shown in Table 1 for all variables considered during model development. Additional data on patient treatment and clinical outcomes not considered for model development are shown in Table 2.

### Univariable Analyses

In univariable (unadjusted) analyses, all acquired and congenital cardiovascular conditions, obesity, other chronic conditions, and treatment of a cardiovascular condition concomitant with COVID-19 treatment were significantly associated with increased odds for severe COVID-19. A total of 167 of 344 patients with single ventricle defects (48.55%) had severe COVID-19, compared with 1051 of 3026 patients with simple BVDs (34.73%) and 728 of 1882 patients with complex BVDs (38.68%). Corresponding unadjusted ORs for all variables including demographics are shown in Table 1.

### Multivariable Mixed-Effects Model

#### Cardiovascular Factors Associated With Severe COVID-19

After controlling for demographics, payer, obesity, other chronic conditions, and treatment for a cardiovascular condition concomitant with COVID-19 treatment during the index visit, we found that both congenital and acquired cardiovascular conditions were associated with increased odds for severe COVID-19. History of cardiac arrest was associated with 892% increased odds of severe COVID-19 (OR, 9.92; 95% CI, 6.93-14.20); cardiogenic shock, with 207% increased odds (OR, 3.07; 95% CI, 1.90-4.96); heart surgery, with 204% increased odds (OR, 3.04; 95% CI, 2.26-4.08); cardiopulmonary disease, with 91% increased odds (OR, 1.91; 95% CI, 1.56-2.34); and heart failure, with 82% increased odds (OR, 1.82; 95% CI, 1.46-2.26) (Table 3).

**Table 3.** Random Intercept Logistic Regression Model of Cardiovascular Factors Associated With Severe COVID-19

Variable	Odds ratio (95% CI)	*P* value^a^
Significant previous or preexisting cardiovascular conditions		
Cardiac arrest	9.92 (6.93-14.20)	<.001
Cardiogenic shock	3.07 (1.90-4.96)	<.001
Heart surgery	3.04 (2.26-4.08)	<.001
Cardiopulmonary disease	1.91 (1.56-2.34)	<.001
Heart failure	1.82 (1.46-2.26)	<.001
Hypotension	1.57 (1.38-1.79)	<.001
Nontraumatic cerebral hemorrhage	1.54 (1.24-1.91)	<.001
Pericarditis	1.50 (1.17-1.94)	.003
Simple BVDs	1.45 (1.29-1.62)	<.001
Venous embolism and thrombosis	1.39 (1.11-1.73)	.006
Other hypertensive disorders	1.34 (1.09-1.63)	.01
Complex BVDs	1.33 (1.14-1.54)	<.001
Essential primary hypertension	1.22 (1.08-1.38)	.002
Cardiomyopathy	0.70 (0.52-0.95)	.03
Atrioventricular and other conduction blocks	0.69 (0.55-0.87)	.002
Nonsignificant previous or preexisting cardiovascular conditions		
Phlebitis and thrombophlebitis	1.41 (0.82-2.40)	.28
Ischemic heart disease	1.24 (0.82-1.89)	.37
Previous intraoperative and postprocedural complications	1.19 (0.81-1.77)	.44
Arrhythmias	1.17 (1.01-1.36)	.06
Chronic rheumatic disease	1.17 (0.87-1.58)	.36
Single ventricle defects	1.06 (0.79-1.41)	.72
Cerebral infarction	1.02 (0.74-1.42)	.88
Lymphatic vessels and lymph node disease	0.93 (0.79-1.09)	.44
Endocarditis	0.93 (0.76-1.14)	.54
Vascular surgery	0.92 (0.83-1.02)	.14
Arterial embolism and thrombosis	0.75 (0.45-1.26)	.35
Complex chronic conditions and comorbidities		
Treated for a cardiovascular condition during COVID-19–related encounter	4.04 (3.22-5.07)	<.001
Gastrointestinal	2.48 (2.25-2.75)	<.001
Neurologic	1.98 (1.83-2.14)	<.001
Malignant neoplasm or cancer	1.66 (1.45-1.90)	<.001
Renal or urologic	1.58 (1.43-1.75)	<.001
Respiratory	1.50 (1.36-1.66)	<.001
Congenital or genetic	1.33 (1.23-1.45)	<.001
Hematologic or immunologic	1.26 (1.14-1.39)	<.001
Obesity	1.25 (1.19-1.31)	<.001
Metabolic	0.98 (0.91-1.06)	.65
Demographic characteristics		
Age		
3-12 mo	0.71 (0.66-0.75)	<.001
13 mo to 2 y	0.60 (0.56-0.64)	<.001
3-5 y	0.73 (0.69-0.76)	<.001
6-11 y	0.91 (0.87-0.95)	<.001
12-17 y	1 [Reference]	NA
Sex		
Female	1 [Reference]	NA
Male	1.35 (1.30-1.40)	<.001
Unknown	1.08 (0.77-1.52)	.67
Race		
American Indian or Alaska Native	1.32 (1.17-1.50)	<.001
Asian or Pacific Islander	1.16 (1.03-1.30)	.02
Black or African American	0.80 (0.76-0.85)	<.001
White	1 [Reference]	
Mixed	0.89 (0.77-1.04)	.21
Other^b^	1.04 (0.97-1.11)	.34
Unknown	0.98 (0.91-1.05)	.65
Ethnicity		
Hispanic or Latino	0.84 (0.80-0.88)	<.001
Not Hispanic or Latino	1 [Reference]	NA
Unknown	1.04 (0.95-1.14)	.47
Payer		
Governmental or public insurance	1 [Reference]	NA
Nongovernmental or private insurance	1.10 (1.04-1.15)	<.001
Unknown	0.96 (0.91-1.02)	.21

Abbreviations: BVDs, biventricular defects; NA, not applicable.

^a^ *P* values were adjusted for multiple comparison using the Benjamini-Hochberg false discovery rate procedure.

^b^ Other was not categorized.

Among 258 patients with previous cardiac arrest, 205 (79.46%) had severe COVID-19, with a mortality rate of 27.91% (72 deaths). Furthermore, 194 of 258 patients (75.19%) with previous cardiac arrest were younger than 12 years.

Similarly, among 124 patients with a history of cardiogenic shock, the rate of severe COVID-19 was 70.16% (87 patients) and the mortality rate was 12.10% (15 deaths). These findings suggest that the increased ORs for these conditions reflected severe morbidity.

Other factors associated with increased odds of severe COVID-19 were hypotension (57% increase; OR, 1.57 [95% CI, 1.38-1.79]), nontraumatic cerebral hemorrhage (54% increase; OR, 1.54 [95% CI, 1.24-1.91]), pericarditis (50% increase; OR, 1.50 [95% CI, 1.17-1.94]), and simple BVDs (45% increase; OR, 1.45 [95% CI, 1.29-1.62]). In addition, venous embolism and thrombosis were associated with 39% increased odds of severe COVID-19 (OR, 1.39; 95% CI, 1.11-1.73); other hypertensive disorders (not including essential primary hypertension), with 34% increased odds (OR, 1.34; 95% CI, 1.09-1.63); complex BVDs, with 33% increased odds (OR, 1.33; 95% CI, 1.14-1.54); and essential primary hypertension, with 22% increased odds (OR, 1.22; 95% CI, 1.08-1.38). Reduced odds for severe COVID-19 were observed for patients with cardiomyopathy and those with atrioventricular and other conduction blocks, which contradicted findings from univariable analyses indicating that these factors were associated with increased odds of severe COVID-19. Other cardiovascular conditions, such as arrhythmias, cerebral infarction, and endocarditis, were not associated with risk for severe COVID-19 in the adjusted analysis. There was also no association between single ventricle defects and increased risk for severe COVID-19 in adjusted analyses.

#### Controlled Variables

Diagnosis of a cardiovascular condition during a COVID-19–related encounter was associated with a 304% increase in odds of severe COVID-19 (OR, 4.04; 95% CI, 3.22-5.07; *P* < .001). All chronic conditions considered except complex chronic metabolic conditions were significantly associated with increased odds of severe COVID-19. Gastrointestinal and neurologic complex chronic conditions had the highest ORs among all chronic conditions that were controlled for in the model (Table 3).

## Discussion

Early in the COVID-19 pandemic, patients with previous or preexisting cardiovascular conditions were observed to be at an increased risk for severe disease when infected with SARS-CoV-2^10,24^; however, to our knowledge, the corresponding magnitude of this association has not been established, especially for pediatric patients. Although the magnitude of the associations found in this study differed considerably across the 26 cardiovascular conditions studied, the results suggest a high overall cardiovascular burden associated with severe COVID-19.

History of cardiac arrest had the highest OR among all variables in the model. Despite the high rate of mortality (27.91%) among pediatric patients with a history of cardiac arrest and severe COVID-19 in this study, only one-third of individuals younger than 12 years were eligible to receive a COVID-19 vaccine or monoclonal antibodies based on recommendations as of September 2021. This suggests that pediatric patients with a history of cardiac arrest are highly susceptible to acute care hospitalization if infected with SARS-CoV-2. Measures to prevent severe illness, such as educating patients to seek timely care and fast-tracking research to make available active (vaccine) and passive (monoclonal antibody) immunizations and new and existing antiviral agents, are crucial. Similar results to those obtained for history of cardiac arrest were revealed for cardiogenic shock.

Previous heart surgery was also found to be associated with severe COVID-19. This finding may be indicative of the cardiovascular strain that accompanies underlying cardiovascular conditions that are severe enough to require surgical intervention. Other acquired cardiovascular conditions, such as cardiopulmonary disease, heart failure, hypotension, nontraumatic cerebral hemorrhage, and pericarditis, were associated with severe COVID-19 (with ORs ranging from 1.50 to 1.91).

In our study, there was a high incidence of severe COVID-19 among patients with any CHD, but an association was found only between BVDs and severe COVID-19. Associations between CHD and other respiratory viruses, such as respiratory syncytial virus, have been described in other studies.^35,36,37,38^ Higher rates of severe influenza in pediatric patients with CHD have been reported to be attributable to limited baseline cardiac and respiratory reserves and limited tolerance for increased respiratory demand in the context of active influenza infection.^35^ In addition, inflammation caused by an active influenza infection may be associated with worsened cardiac function.^35^ In children who contract respiratory syncytial virus–related lower respiratory tract infections, CHD impairs the ability to increase cardiac output, resulting in decreased oxygen delivery.^36^ In pediatric patients, underlying hemodynamically significant CHD has also been associated with risk of severe respiratory syncytial virus–related hospitalization and need for intensive care^37^ as well as substantial disease burden, morbidity, and mortality.^36,38^

The cardiovascular conditions considered in this study may not occur independent of each other, indicating potential additive risk because of the overlapping effects of multiple conditions. For example, cardiogenic shock may result from heart failure, which was associated with increased odds of severe COVID-19 in our study. The presence of other chronic conditions may also be associated with increased morbidity from COVID-19. These considerations are important considering our findings that cardiomyopathy and atrioventricular and conduction blocks were associated with reduced odds of severe COVID-19. This finding must be interpreted with caution, and more granular analyses will be necessary to determine the underlying mechanism and whether angiotensin-converting enzyme II (ACE2) may play a role. Of note, all cardiovascular conditions considered were associated with increased odds of severe disease in the unadjusted (univariable) analysis. Patients with cardiomyopathy and/or atrioventricular and conduction blocks may have comorbidities associated with risk of severe disease.

The association between cardiovascular disease and severe COVID-19 in pediatric patients may be partially explained by the mechanism of SARS-CoV-2 infection. SARS-CoV-2 enters its host cells via receptors for ACE2.^14,39,40^ ACE2 receptors are found in many tissue types, including type II alveolar cells, cardiomyocytes, cardiac fibroblasts, and coronary endothelial cells.^3,41^ ACE2 is an enzyme that converts angiotensin II into angiotensin-(1-7), which plays a role in both local and systemic blood flow regulation.^39^ Specifically, angiotensin-(1-7) helps counteract the inflammatory response to SARS-CoV-2 infection by promoting mild vasodilation and downregulating cytokines, leukocytes, and fibrosis. The specific role of ACE2 in severe COVID-19 remains to be further elucidated. High ACE2 levels have been observed in individuals with mild COVID-19 symptoms.^3,40^ ACE2 expression decreases with age, which could partly explain why the pediatric population has generally had lower prevalence of severe COVID-19 than older adults.^3,5,40^ However, because ACE2 is highly expressed throughout the cardiovascular system, these receptors could be used to directly infect cardiomyocytes and thus cause the myocardial injury and other cardiac effects associated with SARS-CoV-2 infection.^5,40^ Because ACE2 expression is also frequently increased in patients with heart failure (a factor found to be associated with severe COVID-19 in our study), it is plausible that this increased expression provides an explanation for the higher incidence of severe COVID-19 among patients with preexisting cardiovascular disease.^14,41,42^ Limited details are currently available on individual cardiovascular conditions, and to our knowledge, there is no indication of how risk differs by type of condition.

### Limitations

This study has limitations. There are several definitions for classifying the severity of COVID-19, and the definition we chose may not have detected patients who developed cardiovascular complications during hospitalization with COVID-19. Furthermore, newer variants of SARS-CoV-2 have emerged since the end of the study period, and systematic analyses must be performed to identify the factors associated with the severe disease caused by a given variant.

Younger patients (born between 2015 and 2019) may have a shorter history of care by virtue of their age, which may result in missing yet-to-be-diagnosed preexisting conditions. When defining obesity, we found data on BMI for 67.29% of patients and scanned *ICD-10-CM* diagnosis codes for the remaining 32.71% of patients. There may have been some patients with elevated BMI for whom data on BMI and relevant diagnosis codes were absent from the medical record.

The data used for this study were deidentified, with encounter dates that were randomly shifted by, at most, 35 days in either direction. Consequently, the exact month of SARS-CoV-2 infection was unknown. Mild cases of COVID-19 may have been missed given the absence of outpatient or clinic visits for COVID-19 in the study. Furthermore, locations of health systems were known only to the US census region level to preserve the deidentification of location, and we cannot guarantee that patients were included across all clinically meaningful regions of the country in relation to local outbreaks that may have been masked at the census region level.

Only patients with COVID-19 who sought care for any reason at the emergency department, urgent care, or hospital were captured in this study. Consequently, the prevalence and magnitude of the associations identified in this study were conditional on the presentation for treatment in these care settings. Patients with MIS-C could not be excluded because the *ICD-10-CM* diagnosis code for MIS-C was introduced after the start of the study period. Automated database identification of MIS-C was nuanced by the complexity of the MIS-C definition.

## Conclusions

In this cohort study, some previous or preexisting cardiovascular conditions, including cardiac arrest, cardiogenic shock, heart surgery, cardiopulmonary disease, heart failure, hypotension, nontraumatic cerebral hemorrhage, pericarditis, and biventricular defects, were associated with increased COVID-19 severity in US pediatric patients. The data presented in this study highlight the need for pediatric health care professionals to provide careful monitoring and timely care to pediatric patients with preexisting cardiovascular disease and SARS-CoV-2 infection. The significant associations identified suggest the importance of including children in the populations that are eligible to receive a COVID-19 vaccine after the safety and benefits of vaccination have been established.

## Notes

Supplement. **eFigure.** Geographical Distribution of Patients by First Digit of US Zip Code of CRWD Version Used in Study **eTable.** Definitions of Congenital Heart Defects
