# Supplementary material for: Association of Congenital and Acquired Cardiovascular Conditions With COVID-19 Severity Among Pediatric Patients in the US
Source: JAMA Netw Open. 2022 May 17;5(5):e2211967. doi: 10.1001/jamanetworkopen.2022.11967 (PMC9115618; doi:10.1001/jamanetworkopen.2022.11967)
Supplement: Supplement. — eFigure. Geographical Distribution of Patients by First Digit of US Zip Code of CRWD Version Used in Study eTable. Definitions of Congenital Heart Defects [file jamanetwopen-e2211967-s001.pdf]

Supplementary Online Content

Ehwerhemuepha L, Roth B, Patel AK, et al. Association of congenital and acquired cardiovascular conditions with COVID-19 severity among pediatric patients in the US. *JAMA Netw Open*. 2022;5(5):e2211967. doi:10.1001/jamanetworkopen.2022.11967

eFigure. Geographical Distribution of Patients by First Digit of US Zip Code of CRWD Version Used in Study

eTable. Definitions of Congenital Heart Defects

This supplementary material has been provided by the authors to give readers additional information about their work.

© 2022 Ehwerhemuepha L et al. *JAMA Network Open.*

eFigure. Geographical Distribution of Patients by First Digit of US Zip Code of CRWD Version Used in Study

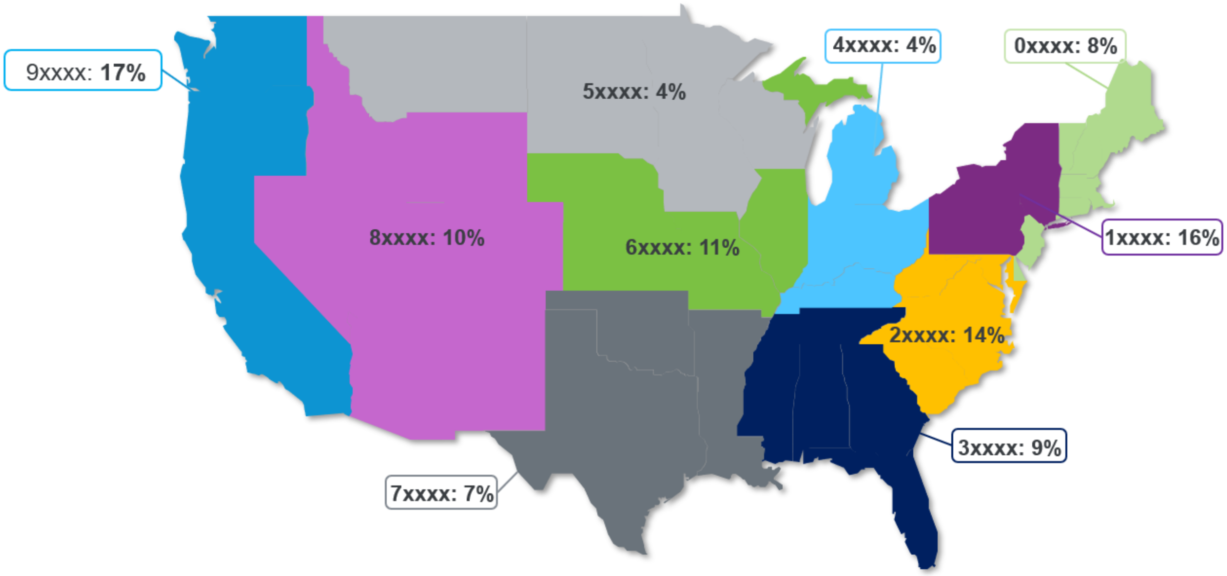

© 2022 Ehwerhemuepha L et al. *JAMA Network Open.*

eTable. Definitions of Congenital Heart Defects
(Adapted, with permission, from Burstein DS, et al., 2019)

**CHD Type**	**Diagnosis**	**ICD-10-CM Codes**
Single ventricle	Common ventricle or double inlet ventricle	Q20.4
	Congenital tricuspid atresia and stenosis	Q22.9, Q22.4, Q22.8
	Hypoplastic left heart syndrome	Q23.4
Simple biventricular	Ventricular septal defect	Q21.0, I27.83
	Ostium secundum type atrial septal defect	Q21.1
	Endocardial cushion defect	Q21.2
	Ostium primum atrial septal defect	Q21.2
	Other bulbus cordis anomaly of septal defect	Q20.8, Q21.4
	Patent ductus arteriosus	Q25.0
	Unspecified defect of septal defect	Q21.9
Complex biventricular	Common truncus	Q20.0
	Complete transposition of great vessels	Q20.3, Q20.8
	Double outlet right ventricle	Q20.1
	L-transposition of great vessels	Q20.5
	Tetralogy of Fallot	Q21.3
	Cor biloculare	Q20.8
	Anomalies of pulmonary valve congenital	Q22.3
	Pulmonary atresia	Q22.0
	Congenital pulmonary stenosis	Q22.1, Q24.3
	Other congenital anomalies of pulmonary valve	Q22.2
	Ebstein's anomaly	Q22.5
	Congenital stenosis of aortic valve	Q23.0
	Congenital insufficiency of aortic valve	Q23.1
	Congenital mitral stenosis	Q23.2
	Congenital mitral insufficiency	Q23.3
	Subaortic stenosis	Q24.4
	Cor triatriatum	Q24.2
	Congenital obstructive anomalies of heart	Q24.8
	Congenital coronary artery anomaly	Q24.5
	Congenital heart block	Q24.6
	Malposition of heart and cardiac apex	Q24.0, Q24.1
	Other congenital anomalies of heart	Q23.8, Q24.8, Q23.9, Q20.9, Q24.9
	Coarctation of aorta	Q25.1
	Interruption of aortic arch	Q25.21

© 2022 Ehwerhemuepha L et al. *JAMA Network Open.*

	Other congenital anomalies of aorta	Q25.4, Q25.41, Q25.42, Q25.43, Q25.44, Q25.45, Q25.46, Q25.47, Q25.48, Q25.49
	Atresia and stenosis of aorta	Q25.29, Q25.3, Q25.2
	Congenital anomalies of great veins	Q26.9, Q26.0, Q26.1, Q26.0, Q26.8
	Total anomalous pulmonary venous return	Q26.2
	Partial anomalous pulmonary venous connection	Q26.3, Q26.4
	Congenital anomalies of pulmonary artery	Q25.71, Q25.5, Q25.6, Q25.8, Q25.9, Q25.79

© 2022 Ehwerhemuepha L et al. *JAMA Network Open.*
